# Novel Bluetooth-Enabled Tubeless Insulin Pump: A User Experience Design Approach for a Connected Digital Diabetes Management Platform

**DOI:** 10.1177/1932296818804802

**Published:** 2018-10-11

**Authors:** Sandhya S. Pillalamarri, Lauren M. Huyett, Aiman Abdel-Malek

**Affiliations:** 1 Insulet Corporation, Billerica, MA, USA

**Keywords:** connected devices, diabetes, insulin pump, internet of things, Omnipod, patch pump, product design, product development, tubeless pump, user experience, user research

## Abstract

**Background:**

Medical device technology is evolving at a rapid pace, with increasing patient expectations to use modern technologies for diabetes management. With the significant expansion of the use of wireless technology and complex, securely connected digital platforms in medical devices, end user needs and behaviors have become essential areas of focus.

**Methods:**

This article provides a detailed description of the user-centered design approach implemented in developing the Omnipod DASH™ Insulin Management System (Insulet Corp., Billerica, MA) Bluetooth^®^-enabled locked-down Android device handheld controller (Personal Diabetes Manager, PDM). Key methodologies used in the PDM design are described, including how the science of user experience (UX) was integrated into new agile product development. UX methods employed included heuristic evaluations of insulin pumps, iterative formative usability testing, information architecture studies, in-home ethnographic visits, participatory design activities, and interviews.

**Results:**

Over 343 users participated in UX research and testing. Key design choices informed by UX research included updating the layout of critical data on the PDM home page, providing access to requested contextual information while a bolus is in progress, and creating an easy-to-understand visual of a 24-hour basal program. Task completion rates for comprehending information on the PDM home page were 87% or greater. The System Usability Scale result for the design prior to limited market release was 84.4 ± 13.4 (out of 100; n = 37).

**Conclusions:**

The UX process described in this article can serve as a blueprint for medical device manufacturers seeking to enhance product development. Adopting UX research methodologies will help ensure that new diabetes devices are safe, easy-to-use, and meet the needs of users.

The rapidly evolving technological landscape has resulted in a significant expansion of the use of wireless technology and complex, securely connected digital platforms in medical devices. Smartphone devices and mobile applications are highly usable and can remove friction from many daily tasks, for example, by providing clear and intuitive driving directions, allowing the deposit and withdrawal of funds, and granting ubiquitous access to information, thus simplifying life. Similarly, people with diabetes can benefit significantly from smartphone devices with applications designed to assist them with the numerous and complex daily management needs, including blood glucose (BG) monitoring, carbohydrate-counting, bolusing for meals, setting reminders and alerts, and adjusting insulin delivery settings, allowing for optimal management and potential avoidance of health complications. These technologies can also provide seamless wireless connectivity and cloud infrastructure for both patients and health care providers to access data to further optimize treatment. To ensure patient safety, usability, and effectiveness with these devices, a rigorous development process that incorporates the science of user experience (UX) is critical.

Smartphone devices equipped with modern touchscreen capabilities, wireless communication radios, and powerful processing powers present a platform that can be adapted to create a safe, secure, and user-friendly handheld controller for an insulin pump. However, the adaptation of a consumer off-the-shelf smartphone for use in a safety-critical connected medical device requires the introduction of key competencies that have not traditionally been a part of medical device development. In addition to the fundamental requirements for mobile software, wireless communications, and cybersecurity, expertise in user-centered design is critically important. In this article we describe the key elements required to create a simple-to-use medical device that leverages smartphone technology, using the remote handheld controller of a recently FDA cleared novel Bluetooth^®^-enabled tubeless insulin management system^1,2^ as an example. This article presents a blueprint for the medical device industry to design products based on smartphone technology that are not only safe and effective, but also easy-to-use.

## Methods

### User Experience in Medical Device Development

#### Usability and Patient Safety

Increased awareness of the frequency and magnitude of medical errors has underscored the importance of considering a medical device’s usability as an integral part of its design. Medical device use-related errors can lead to patient injury and even death.^3,4^ The risk of a use-related error is increased by a poorly designed device with a complicated and difficult UI, which may present problems even for trained users.^3,5,6^ The importance of product usability has been recognized by the FDA, which has added specific usability requirements to its Good Manufacturing Practice regulations and published guidelines for interface design and usability testing.^7-11^ Accordingly, a critical area of focus for medical device manufacturers is the development of products that are designed to balance the end user experience, evaluated against use-risks identified through human factors (HF) analysis and testing.^3,10,12^

#### Novel Bluetooth-Enabled Tubeless Insulin Management System

**Figure 1.**
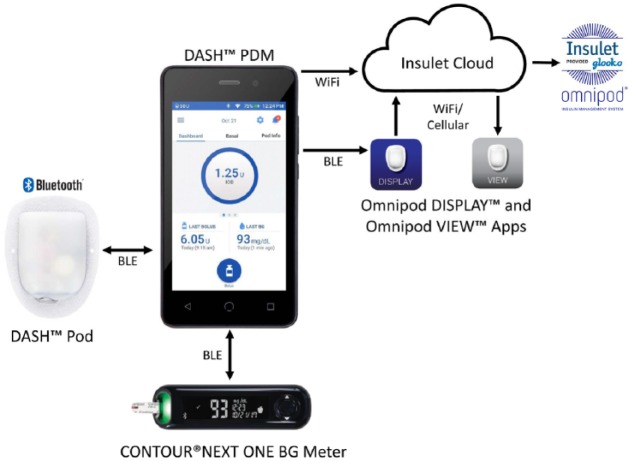
Omnipod DASH™ Personal Diabetes Manager (PDM), Pod, and integrated data communication systems. The PDM communicates with the Pod and the CONTOUR^®^ NEXT ONE Blood Glucose meter through Bluetooth^®^ wireless technology. The PDM uploads data to the secure Insulet Cloud via Wi-Fi, which can then be viewed on a personal cell phone using the Omnipod VIEW™ mobile application. The PDM can also communicate through Bluetooth wireless technology to the Omnipod DISPLAY™ mobile application installed on a personal cell phone. The Omnipod DISPLAY mobile application can then automatically upload data to the Insulet Cloud using Wi-Fi or cellular data. Data uploaded to the Insulet Cloud will automatically merge with the Glooko^®^ data management system to allow integrated data management. Reprinted with permission.^2^

The Omnipod DASH™ Insulin Management System (Insulet Corporation, Billerica, MA) comprises two components: the tubeless insulin pump (Pod) and a wireless remote handheld controller, the Personal Diabetes Manager (PDM) (Figure 1). The system is described in detail elsewhere.^1,2^ Briefly, the PDM is a touchscreen, locked-down Android device that is used to remotely control insulin delivery and periodically monitor Pod status. The PDM device establishes secure, bidirectional, wireless communication with both the Bluetooth-enabled Pod and the CONTOUR^®^ NEXT One BG meter (Ascensia Diabetes Care, Basel, Switzerland). The PDM can also communicate wirelessly with the user’s mobile phone and the Insulet Cloud, enabling applications including Omnipod DISPLAY™ and Omnipod VIEW™, which will allow users and caregivers, respectively, to view a user’s PDM data on their mobile phones (Figure 1).

The genesis of this newly FDA cleared insulin management system was extensive voice-of-user research and input regarding patient needs to transition to pump therapy from multiple daily injections (MDI). To ensure innovative product design and usability and optimize the holistic experience of end users, the UX process was included in each phase of the product development lifecycle. The UIs for the PDM and its related suite of mobile applications were developed considering all edge case scenarios and conditions required for a multilayer connected device system for insulin delivery.

#### Process Overview

At its core, UX is about building systems that are highly usable, safe, and effective while exceeding user expectations, which is achieved by incorporating the voice of the end user throughout all stages of development. The UX process involves four primary phases: user research, conceptualizing, designing, and testing.^13^ The user research phase is paramount as it enables the product development team to unearth and understand unmet user needs, thereby identifying the current state of the user journey and pinpointing breakdowns and pain points. The opportunities and insights discovered during user research drive user-centered design innovation. The conceptualizing phase involves synthesizing identified user needs into documented user and system requirements, which helps the team visualize solutions. Target user groups are identified, researched, and analyzed with cluster mapping and pattern analysis to create archetype user personas that represent a summary of the types of end users who might directly or indirectly influence and experience the end product.^14-17^ The design phase includes brainstorming, white boarding, conceptualizing, and sketching ideas. These ideas are then converted to low-fidelity and high-fidelity screens, which are prototyped rapidly. In the testing phase, robust prototypes that simulate the commercial product are evaluated by potential users recruited and screened for eligibility based on the aforementioned user research persona mapping and market segmentation analysis. The four phases are iterated until all requirements are satisfied and success criteria are met.

#### Team Roles and Responsibilities

While the UX process employs proven scientific methodologies,^10,13,18,19^ it takes a carefully planned team structure, along with cross-functional collaboration, to successfully support this process. For the PDM UI development, core competencies were established around UX research, interaction design, visual design, prototyping, and technical writing. The user research and interaction design teams focused on information architecture, while the technical writing team executed on content strategy and analysis of user’s language within the UI. Working together with the clinical, commercial, training, and software engineering groups, these core UX disciplines delivered on final UX flows, assets, and graphical UI specifications.

### User Experience Methodologies for New Device Development

**Figure 2.**
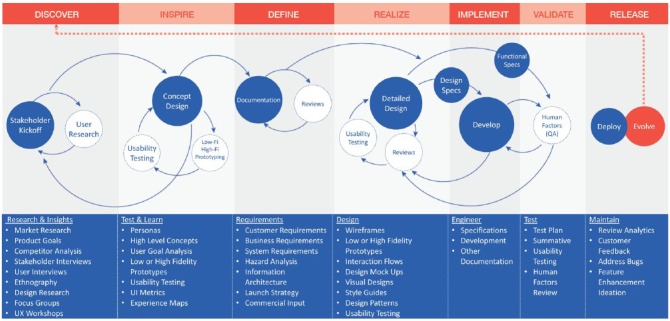
User experience (UX) process implemented and refined for the development of a novel Bluetooth^®^-enabled tubeless insulin management system. The customer requirements specifications (CRS) and product requirements specifications (PRS) naturally help the team create task flows and analysis that is then used to build the use-error risk assessment. These all serve as initial input to the UX team and help drive focused wireframe generation and conceptualization of features, flow and functionality. The UX team simultaneously conducts user research to help inform these requirements and weave those insights gathered into the concepts. The team also continuously tests the concepts while developing moderator guides (that test the use-risk-identified portions of the user interface [UI]), creating highly robust prototypes, and conducting usability testing, card sorts, tree test studies, and others. The UX team finalizes approved designs into detailed annotated UX flows, graphical requirement specifications (GRS), style guides and responsive specifications, among other deliverables. The feedback loop continues as UX performs quality assurance tests on software release builds and logs issue tickets for the development team and ensures final successful implementation of the UI specifications.

The following UX methodologies and techniques were applied in conjunction with the agile product development lifecycle of the PDM (Figure 2).^13,20,21^ The overarching goal was to understand the user journey, frame the users’ motivations and needs in each step of the journey, and create design solutions that are appropriate for each. Representative users within each identified user group were interviewed to create relatable persona snapshots highlighting demographics, behaviors, diabetes management challenges, product success factors, needs and attitudes, and tolerance toward key features such as BG meters, technology, and connectivity.^14-17^ The UX team then visualized the device ecosystem by evaluating the value proposition against that of industry standards to understand best practices, determine what worked well among those standards, and identify opportunities to innovate.

#### Quantitative and Qualitative Measurement

The UX team established key performance indicators (KPIs) such as time on task, number of steps and taps to perform tasks, and ease-of-use measures such as System Usability Scale (SUS) scoring.^19,22-24^ The KPIs were then measured throughout device development and analyzed at each phase to compare the scores to direct user quotes and task completion success, allowing for continuous improvement at each phase.

#### Heuristic Evaluation

In a heuristic evaluation, a team of expert UX professionals examines various aspects of the UI design and assesses them against a set of design principles (heuristics) to help identify areas of concern and opportunities for innovation. The UX team conducted a heuristic evaluation of the PDM UI at the beginning of the development cycle and cataloged findings when comparing the system against ten sets of established UX heuristics: visibility of system status, match between system and the real world, user control and freedom, consistency and standards, error prevention, recognition rather than recall, flexibility and efficiency of use, aesthetic and minimalist design, help users recognize, diagnose and recover from errors, and, finally, help and documentation.^25-28^

#### User Research

A challenge in developing a connected digital platform for diabetes treatment is ensuring its usability across a wide user spectrum with varying needs. How does one design an innovative device that works as well for a 2-year-old child as it does for a 78-year-old adult? User research is therefore arguably the most important phase when building complex systems, as it provides information about the users, their behavior, goals, motivations, and needs. This can be accomplished through focus group and participatory design sessions, in-home visits, and 1-1 user interviews.^10,17,29^ User research for the PDM included hundreds of hours spent with users understanding how they currently use insulin pumps and unearthing MDI users’ concerns and workflows.

#### Information Architecture

Information architecture studies enable the creation of a system content taxonomy that is intuitive and matches user expectations.^19,30,31^ In card sort studies, users are asked to organize cards containing pieces of UI content such as headings, sub-menus, terms, and specific information into categories based on where they would expect to find the content, and to label each category with a name of their choice.^31^ Tree test studies involve the testing and analysis of the various pathways users attempt to find information and successfully complete certain tasks within the UI.^19,30^ Results from iterative card sort and tree test studies were analyzed to update the PDM UI content structure.

#### Rapid Prototyping and Iterative Usability Testing

Lean UX research techniques^21,32^ such as lightning labs were employed during the early stage of PDM development, before employing iterative usability testing within each sprint (a time period of fixed duration with effort focused on specific functionalities of the product). Lightning labs are a custom-developed process involving an intensive week of design iteration during which a cross-functional team works collaboratively on a design challenge and iterates ideas with users. Rapid prototyping allowed for steady input of insights throughout the development process. Tools such as InVision, Android Studio, and Framer were utilized to code prototypes in xCode and Javascript to develop lean and robust prototypes used for both usability and HF testing.

#### Human Factors Evaluation

For medical devices, the HF process is used to first minimize use-related hazards and risks (formative testing), and then confirm that these efforts were successful and users can use the device safely and effectively (summative testing).^9,10,33,34^ The UX team worked closely with the HF team to ensure that identified use risks were mitigated and tested with formative and summative HF evaluation studies. Robust prototypes built by the UX team were tested during formative HF evaluations. The final device programming and summative protocols were adjusted according to findings until all use risks were handled within the UI in a safe and effective manner.

## Results and Discussion

### User Experience Methodologies Applied

#### User Research, Conceptualizing, Design, and Testing

The PDM was developed with frequent input from participants representing a broad variety of potential user groups, including: tubed and tubeless insulin pump users, MDI users, expectant mothers with gestational diabetes requiring insulin, caregivers, nurses, and other identified user groups of various demographics such as age, socioeconomic factors, and location.

The UX team conducted heuristic evaluations of the Omnipod^®^ Insulin Management System (Insulet Corporation, Billerica, MA) and other commercially available insulin pumps, identifying over 55 opportunities for innovation. Iterative UX formative usability testing was conducted, with a total of 8 rounds completed. In addition, 6 in-home ethnographic visits were conducted to observe and understand the needs of insulin pump users with type 1 diabetes (T1D). Three rounds of participatory design sessions were held including a total of 69 participants. The team conducted 1-1 interviews of over 25 people living with diabetes to help build the persona groups. Examples of the personas and their associated goals and diabetes management challenges are shown in Table 1. In total, over 343 users participated in UX development of the system.

**Table 1.** Examples of Personas and Their Associated Goals and Diabetes Management Challenges.

Persona	Goals	Diabetes management challenges
**Tom—**Basic pump user 52 years old, HVAC technician, MDI user for 29 years with T1D who recently switched to the Omnipod^®^ System	• Keep A1c low to forestall health problems • Stay healthy to watch his granddaughter grow up	• Discreetly and easily manage diabetes while driving for work • Manage lows when doing physical work • Afraid to try temporary basal feature his doctor recommended
**Darren—**Driving teen 17 years old, student, sports-loving teenager trying to balance managing T1D with fitting in with peers	• Earn soccer scholarship for college • Fit in with friends • Keep mother from questioning him about his BG highs and lows	• Controlling BG with hormone changes • Dosing insulin during a soccer match, particularly adrenaline highs to third quarter crashes
**Maureen—**Mother of an 11-year-old child with diabetes 42 years old, paralegal, single mother balancing work and caring for her daughter’s T1D	• Manage her daughter’s BG • Teach her daughter to carb count • Give daughter the freedom to be a “normal” kid without excessive monitoring	• Tracking daughter’s BG while she is at school and directing her or the school nurse for treatment • Unexpected and unexplained BG highs, with concerns about CGM accuracy • Getting up to check/treat BG overnight

Abridged excerpts from three example personas developed based on user research.

**Figure 3.**
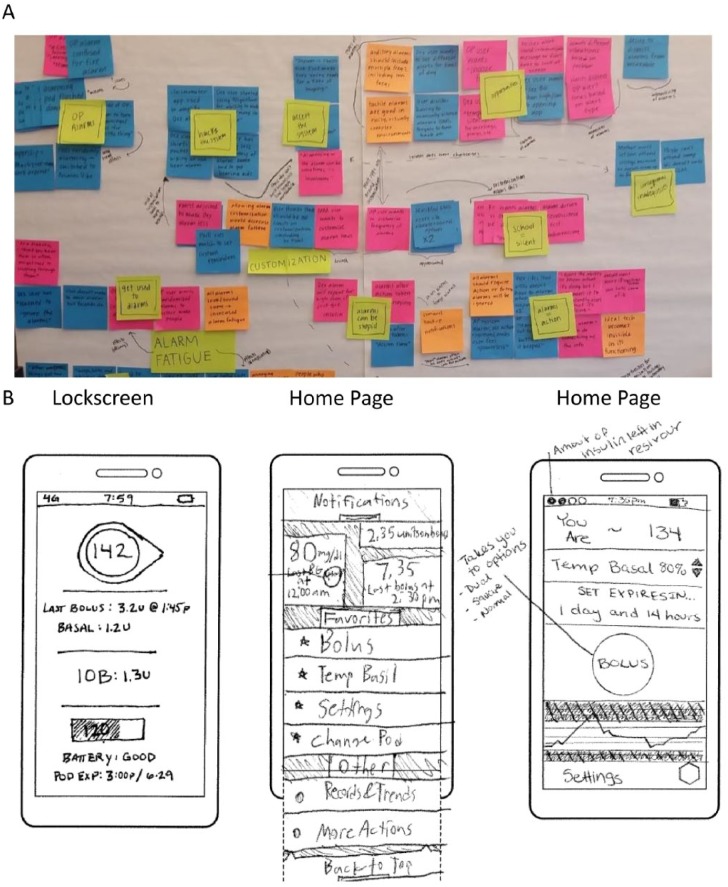
Example images from a participatory design session with insulin pump users and caregivers for the development of a novel Bluetooth^®^-enabled tubeless insulin management system. (A) Participants were given background information and task-based walkthroughs of scenarios, and were asked to write down features, functionalities, and pain points that would affect device usage. These notes were placed on the wall for discussions and the top functionality changes and enhancements were voted and grouped together. (B) The participants were provided stencils of the exact PDM form factor and art supplies and were asked to design the PDM interface that would meet their needs per scenario. Elements from the user-designed interfaces, such as the prominent display of insulin on board (IOB) on the lock screen, the status bar containing insulin reservoir and battery life, and the prominent bolus button and display of last bolus and last blood glucose (BG) on the home page, are shown in the final designs in Figure 4.

**Figure 4.**
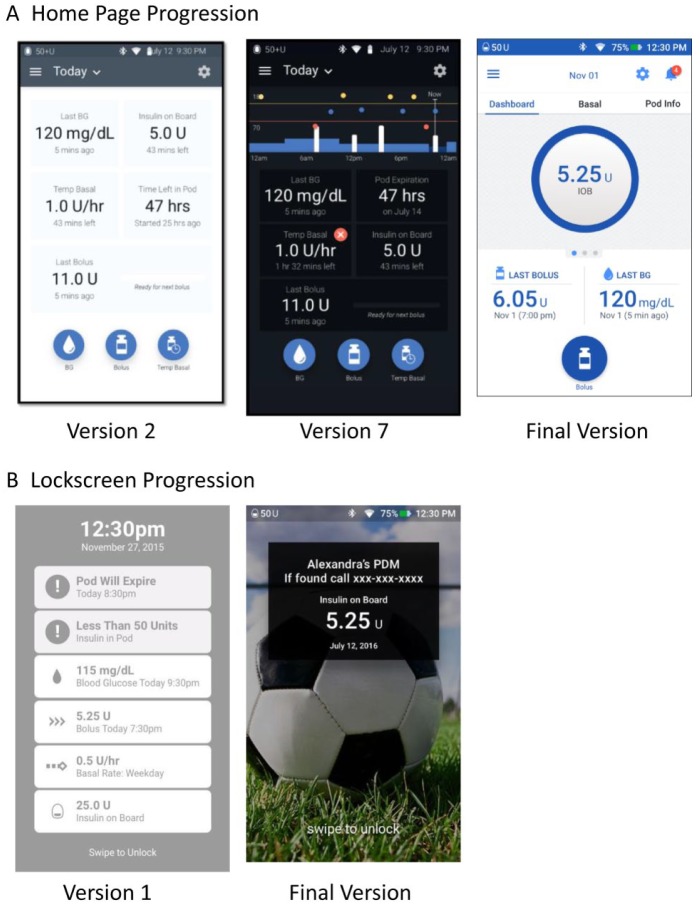
Examples of the PDM home page (A) and lock screen (B) interface designs at various points during the development process. The progression of feedback from end users on early concepts tested through iterative usability testing and other user research methods led to the final design, which has been thoroughly vetted with end user feedback.

Results from one participatory design session are shown in Figure 3. Participants were provided with background information and task-based walkthroughs of diabetes management scenarios, and were asked to write down features, functionalities, and pain points that would affect product usage. These notes were placed on the wall for discussion, mapped and grouped together by theme, and the top functionality changes and enhancements were determined by majority vote, thus enabling prominent user needs and wants to emerge (Figure 3A). The participants were provided stencils of the exact size of the PDM form factor and art supplies and were asked to pictorially design the interface that would meet their needs per scenario (Figure 3B). Elements from the user-designed interfaces (Figure 3B), such as the prominent display of insulin on board (IOB) on the PDM lock screen, the status bar containing insulin reservoir and battery life information, and the prominent bolus button and display of last bolus and last BG on the PDM home page, can be seen in the final UIs in Figure 4. These and other activities were repeated several times throughout the PDM development with design changes made iteratively until a cohesive and complete flow was finalized to meet user needs. Commonly requested features and themes identified, and how these were addressed in the final design, are summarized in Table 2.

**Table 2.** Examples of System Features and Functionality Incorporated Into Omnipod DASH™ Based on User Research and Feedback.

System	User-requested feature	Implemented in Omnipod DASH™
PDM	Personalization of PDM lock screen	Personal lock screen background images and customized message such as name and phone number
	Display amount of insulin in reservoir at all times	Exact amount of insulin in reservoir displayed after the Pod reaches <50 U due to hardware properties
	Display essential information during bolus delivery	IOB amount, last recorded BG, and delivery progress bar displayed during bolus delivery
	PDM functionality on personal smartphone	The Omnipod DISPLAY™ App mirrors the PDM user interface, allowing PDM data to be viewed on personal smartphone
	Food database for bolus calculator	Embedded food library with over 80,000 branded and unbranded products, which integrates directly with the bolus calculator
	Intuitive user interface	Efficiency, usability, and ease of use were development priorities
	Small PDM	The PDM has a small, light-weight form factor comparable to a typical iPhone SE device
Alarms and notifications	Adjustable PDM volume	Volume is adjustable with hard keys on the sides of the PDM. Vibration setting is an option. Hazard alarm tones override volume settings to meet safety requirements.
	Escalating alerts and notifications	Certain alarms provide early notifications prior to a hazard alarm. If alarm action is not performed, a hazard alarm occurs and the PDM will vibrate and tone.
	Ability to snooze alarms with one touch	Alarms and notifications are easily visible on the lock screen. Additional actions are needed to silence an alarm.
BG/CGM	Wireless integration with Dexcom CGM for trend display	The Omnipod DISPLAY App’s iOS Widget allows CGM data to be viewed on the same screen as Omnipod DASH data on the user’s personal smartphone
	Wirelessly integrate with fingerstick BG meter	The PDM receives BG measurements from interoperable BG meter through Bluetooth^®^ wireless technology
Smartphone companion app	App for caregiver to track patient data	The development of the Omnipod VIEW™ app addresses this need

Abridged list of requested features identified during user research and testing and addressed in the final PDM or associated suite of mobile applications.

To gain a better understanding of real-life situations in which the device would be used, the team created mood boards and storyboards to capture the user journey for the identified personas. For example, the team analyzed the series of events that occurs when a young teen living with T1D (Table 1, Darren persona) experiences a Pod failure during class (Figure S1, Supplementary Data). From this exercise, the overall theme identified was that this user persona would prefer for the alarm to notify him privately before sounding an audible alarm, allowing him to manage the situation without drawing unwanted attention to himself. This need, which was echoed by users in participatory design sessions, was addressed in the final design (Table 2, “Alarms and notifications”). Creating a detailed breakdown of the task analysis by outlining required steps, information, and actions to give oneself a bolus, for example, helped the UX team and the software engineering team understand the information flow.

#### Information Architecture Studies

There were 4 rounds of information architecture studies, with an average of 83 participants per round, where users were asked to provide feedback on how well information flowed within prototype versions of the PDM UI. This was done through card sort studies conducted for the overall menu tree structure (60 cards), with studies also conducted on sub-menus for the food library and settings. Users were asked to sort content cards into categories, or explain where they would expect to find specific content and why.

**Figure 5.**
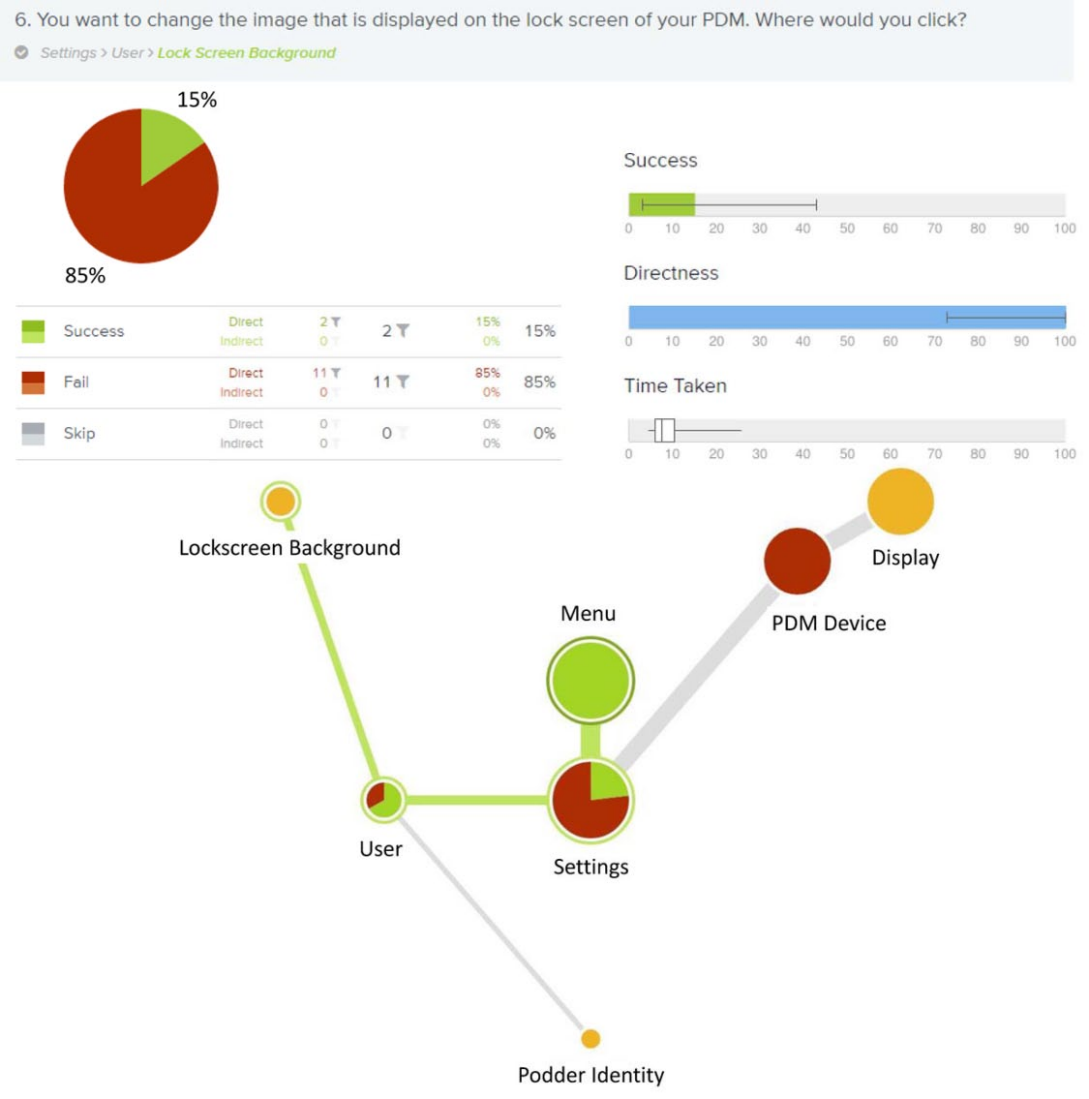
Information architecture studies were a critical component of the system development. Iterative testing and improvement was required to ensure successful completion of critical tasks. This figure shows a depiction of a card sort testing analysis of the early menu structure. Participants were asked where they would go to change the background theme image for the PDM lock screen. During the early development phases, the information architecture was confusing to users as indicated by the red color showing failure to find the lock screen image. Tests such as these provided necessary guidance and data to help structure the navigation and flow of information within the system.

In one example (Figure 5) participants were asked where they would go to change the background theme image for the PDM lock screen. During the early development phases, the information architecture was confusing to users, with a failure rate of 85% to find the lock screen image. Iterated card sort and tree test studies provided necessary guidance and data to structure the navigation and flow of information within the UI, ensuring desired information could be found by the user quickly and with low risk of failures.

#### Developing the User Interface

Working closely with the software engineering team within an agile engineering process, use case and scenario mapping helped organize a prioritized list of UX features and tasks to be completed into multiple feature sets that allowed time for the full process of UX to occur within each focused development period. User input and testing helped refine the style guide, form and input fields, graphs, scroll wheels, and other interactive and visual UI elements.

Upon completion of UX research and testing, the team developed detailed UX flow diagrams along with final graphical UI specifications for the final PDM evaluated and submitted to the FDA. The same process was repeated for the suite of associated mobile applications.

### Implications to the Final Design

Information learned from the iterative UX research process was used to inform key PDM design choices. Table 2 presents examples of commonly requested features, and how these were addressed in the final design. Additional design choices based on user feedback are described below.

**Figure 6.**
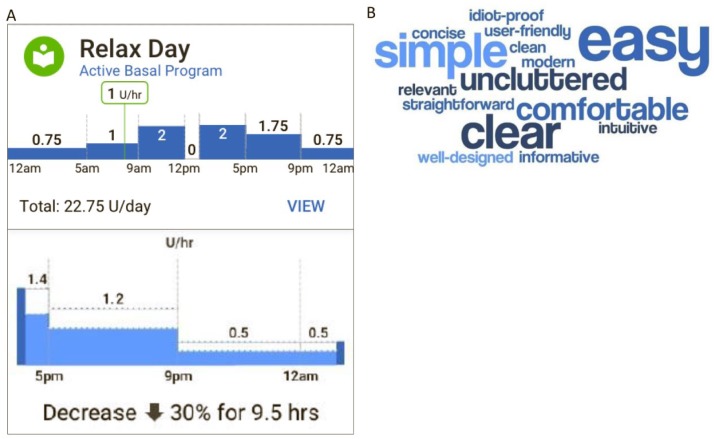
Design changes based on user feedback included updating the visual display of information on the PDM home page and providing a pictorial depiction of basal insulin delivery. (A) Images showing the basal rate graph (top panel) and a zoomed-in view of the temporary basal rate graph (bottom panel) that stemmed from user insights, which showed that users wanted a pictorial depiction of insulin delivery, rather than solely showing the program with numbers and tables. The basal rate graph appears in the “Basal” tab of the home page and when creating or editing basal programs, activating temporary basal rates, and creating temporary basal presets. (B) Test participants were asked to choose three words or phrases that best described their experience using the PDM home page concepts (including the “Dashboard,” “Basal,” and “Pod Info” tabs) during the development phases of the user interface. This word cloud was generated directly from 9 users’ quotes and was used to represent and understand user satisfaction with the home page, with words appearing with a higher frequency presented in larger font.

The PDM home page design was iterated until users were satisfied with the layout of critical data and the visual design delighted them (Figure 4A). The dark background was changed to light for the final version, as users expressed difficulties with the dark background. A notification icon, with a badge indicating the number of new notifications, was added to provide a clear and consolidated indication of any alerts or alarms throughout the system. An easy-to-understand visual representation was created for a 24-hour basal program and for a change in basal rates for a temporary basal rate increase or decrease (Figure 6A). The basal rate graph appears in the “Basal” tab of the home page and when creating or editing basal programs, activating temporary basal rates, and creating temporary basal presets. Task completion rates for comprehending information on the home page (including the “Dashboard,” “Basal,” and “Pod Info,” tabs) were 87% or greater (a typical target is 70%). User satisfaction with the home page concepts was captured by “The top 3 words you would use to describe this page,” shown by the word cloud in Figure 6B, which provides a concise visual depiction of the users’ impressions of the system based on frequency of word use.

Simplicity in the display of information was a main goal in the design development of the PDM UI. One key change from earlier concepts of the PDM lock screen (Figure 4B) was the simplification of information displayed. Earlier concepts displayed information on several data points and metrics including notifications, last BG, last bolus, and current basal rate, all with equal weight. The final version displays the IOB prominently, as this was the information users were most interested to see at a glance, with reservoir insulin and PDM battery life visible in the status bar. Personalization of the lock screen was added, including an image and a customized message that may be used for name and contact phone number.

The number of button presses/taps was optimized for commonly performed tasks, such as programming a meal bolus with manual entry of a carbohydrate amount and setting a temporary basal rate decrease. Access was provided to requested contextual information (IOB, last bolus) while a bolus is in progress (Table 2).

The design was evaluated prior to the limited market release. The mean SUS^22-24^ score was 84.4 ± 13.4 (out of 100; n = 37).

### Human Factors Results

The HF validation test was a simulated-use study structured to mimic actual use, utilized an equivalent to production version of the system, and was designed to be sufficiently sensitive to capture use related problems, if any existed. Participants were representative of actual users. Task-based scenarios focused on the highest priority tasks associated with insulin delivery and represented those that a user would complete during typical everyday use (examples include activating a new Pod, delivering a bolus using the food library, and responding to critical alarms). All use-related issues that occurred during testing were evaluated through root cause analysis to determine the failure mode, root cause of the failure, consequence of the failure, and mitigations that exist to reduce the frequency of occurrence and risk associated with the failure.

The results of the validation test demonstrated that the insulin management system was safe and effective for the intended users, uses, and use environments. Any additional modifications to the UI related to the safety critical tasks (including the device, training, and labeling) would not further reduce risk, were not possible, or were not practical, and the remaining residual use-related risks are outweighed by the benefits derived from use of the device. Moreover, a process has been established for PDM software updates to be completed by the user as required, with usability and HF testing being completed prior to implementing this functionality.

## Conclusion

A rigorous UX research and design process is foundational to the development of diabetes medical devices to ensure safety, effectiveness, and ease of use and reduce the daily burden of managing diabetes. The development of the Omnipod DASH PDM included UX design and research in each step of the product development lifecycle. The UX process developed for this system can serve as a blueprint for other diabetes device manufacturers seeking to improve usability of their devices.

## Supplemental Material

Pillalamarri_Supplementary_Data – Supplemental material for Novel Bluetooth-Enabled Tubeless Insulin Pump: A User Experience Design Approach for a Connected Digital Diabetes Management Platform Click here for additional data file. Supplemental material, Pillalamarri_Supplementary_Data for Novel Bluetooth-Enabled Tubeless Insulin Pump: A User Experience Design Approach for a Connected Digital Diabetes Management Platform by Sandhya S. Pillalamarri, Lauren M. Huyett and Aiman Abdel-Malek in Journal of Diabetes Science and Technology
