# Supplementary material for: Novel Bluetooth-Enabled Tubeless Insulin Pump: A User Experience Design Approach for a Connected Digital Diabetes Management Platform
Source: J Diabetes Sci Technol. 2018 Oct 11;12(6):1132–42. doi: 10.1177/1932296818804802 (PMC6232743; doi:10.1177/1932296818804802)
Supplement: Pillalamarri_Supplementary_Data – Supplemental material for Novel Bluetooth-Enabled Tubeless Insulin Pump: A User Experience Design Approach for a Connected Digital Diabetes Management Platform [file Pillalamarri_Supplementary_Data.docx]

**­­Novel Bluetooth®-Enabled Tubeless Insulin Pump: A user experience design approach for a connected digital diabetes management platform­­**

Sandhya S. Pillalamarri, BS, MHCI, ALM, Lauren M. Huyett, PhD, Aiman Abdel-Malek, PhD

**Supplementary Data**


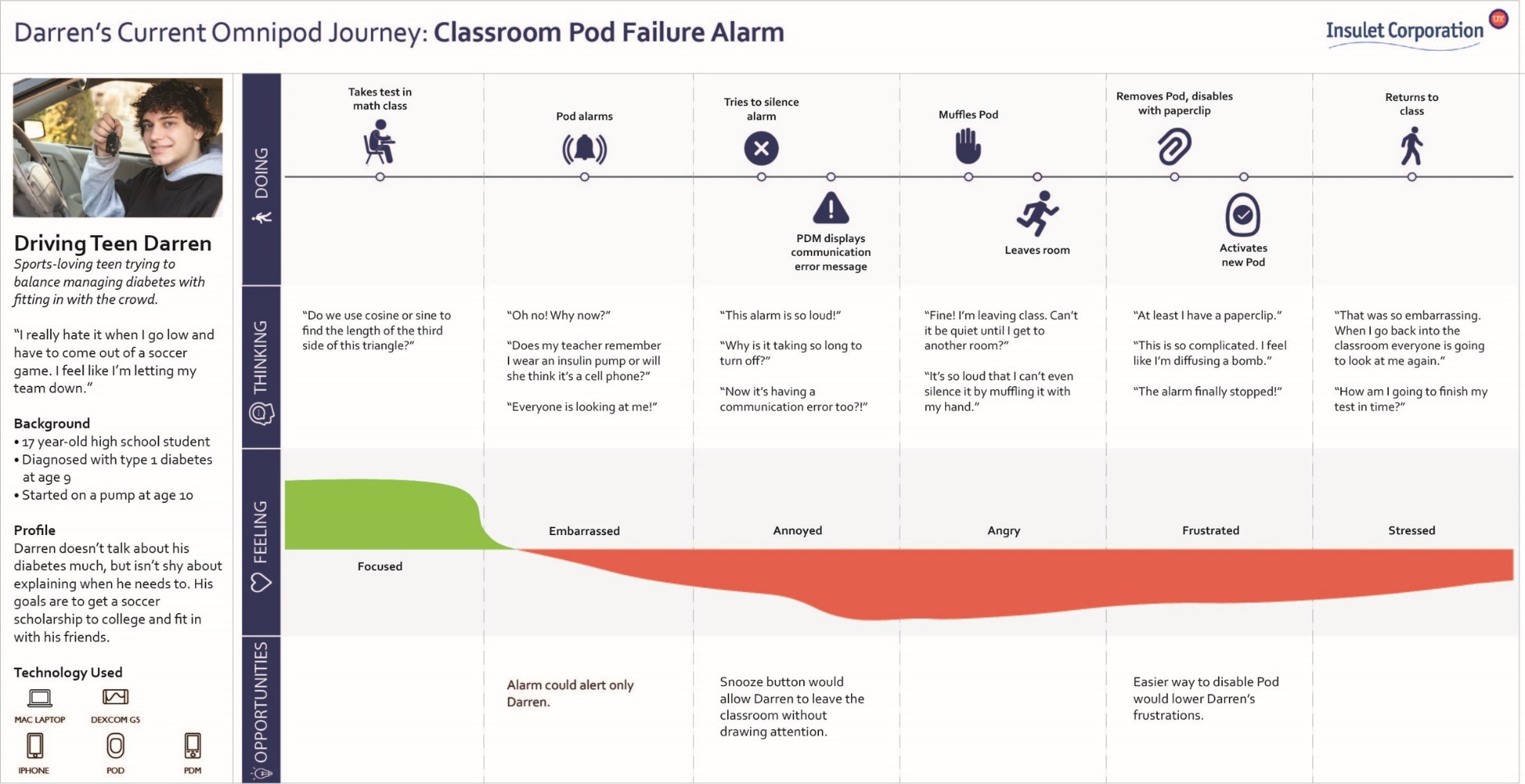
 Figure S1. Example of a user journey for a specific persona and scenario with actions, thoughts, feelings, and opportunities for innovation. In this scenario, the team conducted contextual inquiries and home visits to better understand the journey and breakdowns of alarms and alerts experience per persona.
